# Effectiveness of informational decision aids and a live donor financial assistance program on pursuit of live kidney transplants in African American hemodialysis patients

**DOI:** 10.1186/s12882-018-0901-x

**Published:** 2018-05-03

**Authors:** L. Ebony Boulware, Patti L. Ephraim, Jessica Ameling, LaPricia Lewis-Boyer, Hamid Rabb, Raquel C. Greer, Deidra C. Crews, Bernard G. Jaar, Priscilla Auguste, Tanjala S. Purnell, Julio A. Lamprea-Monteleagre, Tope Olufade, Luis Gimenez, Courtney Cook, Tiffany Campbell, Ashley Woodall, Hema Ramamurthi, Cleomontina A. Davenport, Kingshuk Roy Choudhury, Matthew R. Weir, Donna S. Hanes, Nae-Yuh Wang, Helene Vilme, Neil R. Powe

**Affiliations:** 1 Division of General Internal Medicine, Duke University School of Medicine, 411 W. Chapel Hill, St Suite 500, Durham, NC 27110 USA; 2 Department of Epidemiology, Johns Hopkins Bloomberg School of Public Health, Baltimore, MD USA; 3 Welch Center for Prevention, Epidemiology and Clinical Research, Baltimore, MD USA; 4 Division of General Internal Medicine, Johns Hopkins School of Medicine, Baltimore, MD USA; 5 Division of Nephrology, Johns Hopkins School of Medicine, Baltimore, MD USA; 6 Department of Surgery, Johns Hopkins School of Medicine, Baltimore, MD USA; 7 Department of Cardiology, University of Washington School of Medicine, Seattle, WA USA; 8 Nephrology Center of Maryland at MedStar Good Samaritan Hospital, Baltimore, MD USA; 9 Department of Biostatistics and Bioinformatics, Duke University School of Medicine, Durham, NC USA; 10 Division of Nephrology, University of Maryland School of Medicine, Baltimore, MD USA; 11 Department of Biostatistics, Johns Hopkins Bloomberg School of Public Health, Baltimore, MD USA; 12 Department of Medicine, San Francisco General Hospital and University of California, San Francisco, CA USA

**Keywords:** Decision aid, End stage renal disease, Financial support, Live donor kidney transplant, Race disparities

## Abstract

**Background:**

African Americans have persistently poor access to living donor kidney transplants (LDKT). We conducted a small randomized trial to provide preliminary evidence of the effect of informational decision support and donor financial assistance interventions on African American hemodialysis patients’ pursuit of LDKT.

**Methods:**

Study participants were randomly assigned to receive (1) Usual Care; (2) the Providing Resources to Enhance African American Patients’ Readiness to Make Decisions about Kidney Disease (PREPARED); or (3) PREPARED plus a living kidney donor financial assistance program. Our primary outcome was patients’ actions to pursue LDKT (discussions with family, friends, or doctor; initiation or completion of the recipient LDKT medical evaluation; or identification of a donor). We also measured participants’ attitudes, concerns, and perceptions of interventions’ usefulness.

**Results:**

Of 329 screened, 92 patients were eligible and randomized to Usual Care (*n* = 31), PREPARED (*n* = 30), or PREPARED plus financial assistance (*n* = 31). Most participants reported interventions helped their decision making about renal replacement treatments (62%). However there were no statistically significant improvements in LDKT actions among groups over 6 months. Further, no participants utilized the living donor financial assistance benefit.

**Conclusions:**

Findings suggest these interventions may need to be paired with personal support or navigation services to overcome key communication, logistical, and financial barriers to LDKT.

**Trial registration:**

ClinicalTrials.gov [NCT01439516] [August 31, 2011].

## Background

For over 10 years, there have been growing efforts to improve patients’ access to living donor kidney transplantation (LDKT). Patient advocacy, professional and government organizations have invested substantial resources [1–5] to support these efforts. Despite this, LDKT rates have plateaued in recent years, underscoring the urgency to establish best practices to improve LDKT access [6]. Inadequate access to LDKT has been especially problematic for African Americans who have had persistently lower rates of LDKT compared to others [7, 8].

African American patients with kidney failure confront numerous barriers to LDKT including poor awareness [9] and knowledge [10] of LDKT, difficulty identifying donors [11–13], as well as financial barriers [14, 15] to LDKT. Evidence shows African Americans are less likely than Whites to be fully informed of their treatment options, including LDKT, before initiating hemodialysis [9, 10]. African Americans have also been shown to be highly sensitive to the financial impact of living kidney donation on potential donors, further limiting their LDKT utilization [14, 16]. Interventions to increase African American patients’ knowledge of LDKT have been widely advocated as important to help address disparities [17, 18]. The National Living Donor Assistance Program [19] also provides financial assistance to low income LDKT donors to help address disparities. While these efforts have been in place for some time, their uptake and effectiveness to improve LDKT rates among African Americans have not been well studied.

Interventions enabling informed decisions about LDKT and providing financial support for LDKT could be particularly useful for African American patients who have recently initiated dialysis, as they may be poorly educated about kidney treatments, unaware of potential financial support, and still in the early phases of making longer-term decisions about LDKT. However, evidence to support the widespread adoption of these interventions does not yet exist. We conducted a small randomized clinical trial among urban African Americans receiving hemodialysis to examine the potential effectiveness of delivering informational decision support and LDKT donor financial assistance to improve their pursuit of LDKT.

## Methods

### Study design

The Providing Resources to Enhance African American Patients’ Readiness to Make Decisions about Kidney Disease (PREPARED) Study was a small, 6-month randomized clinical trial. The intent of PREPARED was to provide rigorous preliminary evidence that interventions might be effective to guide the conduct of larger national trials. We enrolled African American patients with end stage renal disease who had recently initiated in-center hemodialysis. We randomly assigned participants with equal probability to receive [1] usual dialysis care in the dialysis facility (“Usual Care”), [2] informational decision aids (i.e., a video and a book describing LDKT and other forms of renal replacement therapy, referred to as “PREPARED information”), or [3] the PREPARED information plus a living kidney donor financial assistance program (referred to as “PREPARED Plus”). Participants could not be feasibly blinded to treatment group after receiving the PREPARED information or financial assistance interventions. Data collectors assessed outcomes using objective measures 1, 3, and 6 months after randomization. The Johns Hopkins School of Medicine Institutional Review Board approved all protocols and consent procedures. The Duke University School of Medicine Institutional Review Board approved all data analysis procedures. The study was registered with ClinicalTrials.gov [NCT01439516]. Study enrollment began in April 2012 and concluded in July 2013. Details of our protocol have been previously published [20].

### Study setting and participants

We recruited participants from 11 outpatient hemodialysis facilities in the Baltimore, MD metropolitan region. Enrollment initiated in April 2012. Research staff confirmed potential participants’ date of dialysis initiation with dialysis nurses, and they administered questionnaires in hemodialysis facilities to assess patients’ study eligibility, including an assessment of their cognitive function (the MiniCog) [21]. Patients were eligible to participate if they initiated hemodialysis within 2 years of the date of screening, spoke English, were age 18 years or older, and had self-reported African American race. We excluded patients with self or hemodialysis nurse-reported dementia, objective cognitive impairment, prior kidney transplant, or potential medical exclusions from receiving LDKT, including cancer within the previous 2 years, advanced congestive heart failure, end stage liver disease, unstable coronary artery disease, pulmonary hypertension, severe peripheral vascular disease, or chronic debilitating infections.

### Enrollment and randomization

Research staff obtained participants’ informed written consent and administered participants a pre-enrollment questionnaire in hemodialysis facilities. Staff then contacted participants via telephone to complete additional pre-enrollment questions and to conduct trial enrollment and randomization after all eligibility criteria were met. A statistician not involved in participant recruitment generated the sequence of blocked intervention assignments using SAS version 9.3, with equal allocation to the 3 study arms within randomly selected block sizes of 3 and 6, stratified by the hemodialysis facilities. Allocation was concealed to research staff enrolling participants until the end of the baseline telephone interview, when participants were formally enrolled. We employed the methods of ‘sequentially numbered opaque sealed envelopes’ for randomization.

### Study interventions

The 45-min PREPARED DVD video described LDKT and other kidney replacement treatment options from the perspectives of patients receiving each treatment and their family members. The PREPARED book was written at a 4th grade reading level and summarized scientific evidence, derived from systematic literature reviews, about differences in treatments’ effects on numerous aspects of patients’ lives, including longevity, morbidity, quality of life and financial matters. The development and content PREPARED decision aids have been described elsewhere [20, 22].

The living donor financial assistance program offered participants’ potential live donors reimbursement up to $1600.00 for qualifying medical and non-medical expenses related to live kidney donor evaluation, donation, or post-donation convalescence (up to 10 weeks post live kidney donor procedures). The program covered a broader array of expenses related to live kidney donation than the National Live Donor Assistance Program [23] and did not require potential donors to qualify based on their personal incomes [19, 20].

### Intervention delivery

Research staff met with participants assigned to receive study interventions at hemodialysis facilities on non-hemodialysis days. Staff oriented participants to the contents of the PREPARED book and video using a script and sat with participants while participants watched the video. Staff encouraged participants to share PREPARED materials with their family members and health care providers. Staff reviewed with participants the features of live donor financial assistance program using a script. Staff referred participants to their health care providers for answers to questions participants had about clinical care, treatment options, or other aspects of treatment decision-making after reviewing PREPARED materials.

Participants assigned to “Usual Care” received their routine medical care in hemodialysis facilities after enrollment. Participants in any of the three groups could have received educational materials or financial assistance through their usual health care.

### Data collection

#### Participant demographic, clinical characteristics, and experiences with hemodialysis care at enrollment

At enrollment, we assessed participants’ sociodemographic characteristics and factors that could affect participants’ likelihood of identifying potential living kidney donors, including family function (Family APGAR index, which measures five aspects of family function) [24] and their perceived financial well-being or distress (using the personal financial well-being scale) [25]. We also assessed participants’ comorbidity (using the Charlson Comorbidity Index [26] modified for end stage renal disease) and their presence of depression (using the validated PHQ-8 [27]). We asked participants how long they had received care in their current hemodialysis treatment facility, and we assessed their satisfaction with care using questions adapted from the Consumer Assessment of Healthcare Providers and Systems (CAHPS®) In-Center Hemodialysis Survey [28]. We also asked participants about their preparation for kidney replacement therapy and/or LDKT, initiation of hemodialysis with a fistula or graft, and their completion of a kidney transplant recipient medical evaluation and/or placement on a kidney transplant waiting list. We further asked participants about their concerns regarding LDKT, rated on a 10-point scale ranging from 0 (no concern) to 10 (extremely concerned).

#### Participant perceived involvement in kidney treatment decisions at enrollment

We asked participants to report their perceived involvement in treatment decisions with the questions, “In the last 12 months, did either your kidney doctors or dialysis center staff talk to you as much as you wanted about which treatment is right for you?” and “In the last 12 months, were you as involved as much as you wanted in choosing the treatment for kidney disease that is right for you?”

### Outcomes

Our primary outcome was participants’ newly self-reported achievement of behaviors reflecting their consideration and/or pursuit of LDKT at 1, 3, or 6 months follow up. Behaviors included patients’: [1] discussion about LDKT with their family members; [2] discussion about LDKT with their doctor; [3] initiation of the recipient medical evaluation for LDKT; [4] completion of the recipient evaluation for LDKT; and [5] identification of a potential live kidney donor. We assessed these LDKT behaviors and any new behaviors via participants’ self report by telephone interviews at baseline and 1, 3, and 6 months after enrollment. We also assessed participants’ beliefs about kidney transplant and their concerns about LDKT.

### Intervention fidelity and perceived usefulness

Study staff documented the occurrence of in-person meetings with participants to deliver interventions. In a small number of instances when participants were not able to meet with study staff, staff mailed or delivered interventions to participants’ homes. We asked participants their views on usefulness of PREPARED materials in their treatment decision making. We also asked eligible participants whether they shared the donor financial assistance program with family members or friends.

### Statistical analysis

We quantified changes in participants’ probability of achieving at least one new LDKT action over 6 months and differences in changes among study groups. The main independent variable was the randomly assigned intervention group. We hypothesized a priori that the PREPARED information intervention alone (designed to overcome educational barriers to LDKT) and the PREPARED information paired with the financial assistance intervention (designed to overcome financial barriers to LDKT) would be more effective at improving participants’ pursuit of LDKT compared to Usual Care. A priori, we estimated a sample size of 210 (70 participants randomized to each group) would provide over 80% power to detect a trend of increasing LDKT pursuit across groups at 6 months [29]. We conducted descriptive cross-sectional analyses (using Fisher’s exact test) to compare differences in participants’ achievement of LDKT behaviors at any time point among groups at baseline and 6 months. We also constructed longitudinal generalized estimating equations (GEEs) to estimate group differences in participants’ probabilities of accomplishing at least one additional LDKT behavior at 1, 3 or 6 months follow up. Models specified an unstructured correlation structure for repeated measures and adjusted for participants’ baseline comorbidity scores, which were not balanced across study groups at baseline. We also modeled changes in participants’ self-reported beliefs regarding transplant and concerns about LDKT at baseline, 1, 3, and 6 months using longitudinal GEEs. We also conducted a sensitivity analysis accounting for potential clustering of findings among participants receiving care in the same hemodialysis facilities. In this analysis, we fit a simple generalized linear mixed model with a random intercept for hemodialysis facility and a random intercept for participant (nested within facility). We performed analyses using R version 3.2.0 (R Foundation for Statistical Computing (2015), Vienna, Austria).

## Results

### Participant screening, enrollment, and retention

**Fig. 1:**
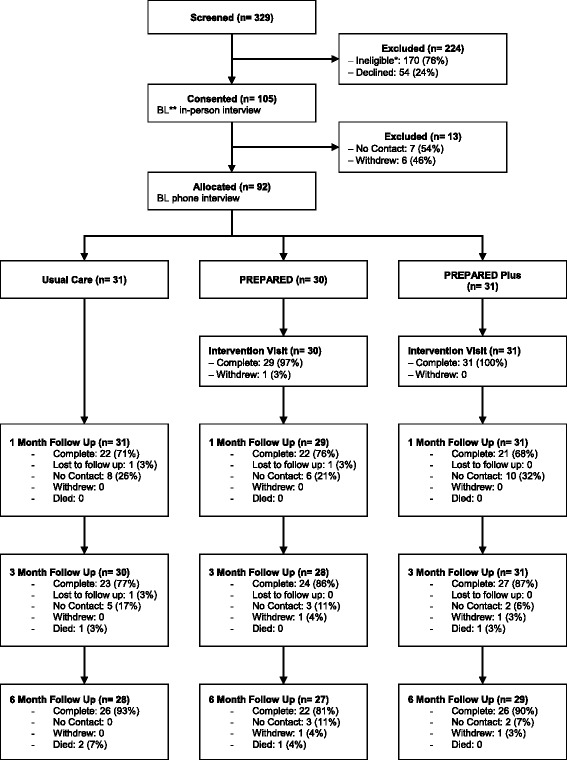
CONSORT Flow Diagram for study eligibility, screening, consent, enrollment, randomization, and follow-up. *Reasons for ineligibility included deceased at time of contact (*n* = 6), previous kidney transplant (*n* = 14), in nursing home or hospice (*n* = 8), medically unstable (*n* = 22), Non-African American (*n* = 18), receiving hemodialysis for longer than the 1–2 year (*n* = 1), no longer on hemodialysis or recovered their kidney function (*n* = 4), need for proxy (*N* = 3), switched dialysis treatment center at the time of contact (*n* = 9), and other (*n* = 2). **BL = Baseline

Between April 2012 and July 2013, we assessed the eligibility of 329 people, of whom 159 were eligible to participate and 54 declined to participate. A majority of potential participants (*n* = 83) were not eligible because they were medically unsuitable for transplant based on the exclusion criteria. Of potentially eligible people, 105 consented and completed a baseline-in-person interview, and 92 completed the enrollment interview via phone. These 92 participants were randomly assigned to receive Usual Care (*n* = 31), PREPARED information (*n* = 30), or PREPARED information plus financial assistance (*n* = 31). Overall, 84%, 73%, and 90% of participants who were originally enrolled completed the 6-month follow-up in the usual care, PREPARED, and PREPARED Plus groups, respectively. Recruitment fell short of our a priori goal (total enrollees 210 planned, 70 participants per group) due to administrative funding cuts limiting our capacity to expand recruitment to additional hemodialysis facilities (Fig. 1).

### Participant characteristics at enrollment

All 92 study participants were of self-identified African American race. Participants were similar with regard to all sociodemographic characteristics. Most (73%) participants had at least a high school education and over half (59%) reported annual household incomes of less than $20,000 annually. Few (13%) were employed full- or part-time, nearly one third were married, and nearly half had at least 1 parent living. Half of participants (50%) had ≥9th grade health literacy. The majority of participants (62%) had initiated dialysis and received care at the hemodialysis unit from which they were recruited within less than 1 year from the time of study enrollment. Median comorbidity scores were statistically significantly greater among participants in the usual care and PREPARED Plus groups compared to participants in the PREPARED information group. Few participants reported advance preparation for LDKT. Nearly half (46%) of all participants reported initiating hemodialysis in the emergency room and a majority (67%) reported initiating hemodialysis with catheter. Few (18%) reported they had completed a kidney transplant recipient evaluation, and a majority reported they were not on a waiting list for a deceased donor kidney. Participants’ median ratings for satisfaction with hemodialysis center staff and facilities were high (Table 1).

**Table 1:** Participant sociodemographic and medical characteristics

	Overall *N* = 92 n(%)^a^	Usual Care *N* = 31 n(%)	PREPARED *N* = 30 n(%)	PREPARED Plus *N* = 31 n(%)	p^a^
Sociodemographic Characteristics					
Age [mean, standard deviation]	53 [14]	52[12]	53[16]	55[13]	0.6
Gender					0.3
Female	47 (51)	13 (42)	15 (50)	19 (61)	
Male	45 (49)	18 (58)	15 (50)	12 (39)	
Highest Level of Education					0.5
High school	67 (73)	21 (68)	23 (77)	23 (74)	
2 years of college	12 (13)	6 (19)	2 (7)	4 (13)	
College graduate	8 (9)	4 (13)	2 (7)	2 (7)	
Graduate/professional school	5 (5)	0 (0)	3 (10)	2 (7)	
Annual Household Income					0.2
≤ $10,000	30 (33)	12 (39)	9 (30)	9 (29)	
> $10,000 to $20,000	24 (26)	8 (26)	10 (33)	6 (19)	
> $20,000 to $40,000	19 (21)	5 (16)	8 (27)	6 (19)	
> $40,000	9 (10)	1 (3)	1 (3)	7 (23)	
Refused/don’t know	10 (11)	5 (16)	2 (7)	3 (10)	
Employment					0.5
Full- or part-time	12 (13)	2 (6)	6 (20)	4 (13)	
Student/homemaker	3 (3)	1 (3)	1 (3)	1 (3)	
Retired	18 (20)	5 (16)	7 (23)	6 (19)	
Retired/disabled	36 (39)	17 (55)	7 (23)	12 (39)	
Other/unemployed	23 (25)	6 (20)	9 (30)	8 (25.81)	
Marital status					0.1
Married	30 (33)	7 (23)	10 (33)	13 (42)	
Separated	3 (3)	1 (3)	2 (7)	0 (0)	
Divorced	15 (16)	8 (26)	1 (3)	6 (19)	
Single/never married	36 (39)	14 (45)	12 (40)	10 (32)	
Widowed	7 (8)	1 (3)	4 (13)	2 (6)	
Refused	1 (1)	0 (0)	1 (3)	0 (0)	
Family composition					
No. of children, median [interquartile range, IQR]	2 [1–4]	2 [2–4]	2 [0–3]	2 [2–4]	0.1
No. of siblings, median [IQR]	3 [1–4]	3 [2–5]	3 [1–4]	3 [2–4]	0.6
At least 1 parent living	45 (49)	13 (42)	17 (57)	15 (48)	0.6
Health insurance					
Private	24 (26)	6 (19)	7 (23)	11 (36)	0.4
Medicare	44 (48)	16 (52)	16 (53)	12 (39)	0.5
Medicaid	41 (45)	16 (52)	12 (40)	13 (42)	0.7
Other/uninsured	11 (12)	3 (10)	5 (17)	3 (10)	0.7
Health literacy					0.1
4th–6th grade	9 (10)	4 (13)	0 (0)	5 (16)	
7th–8th grade	27 (29)	6 (19)	13 (43)	8 (26)	
≥ 9th grade	46 (50)	15 (48)	15 (50)	16 (52)	
Poor vision, could not complete	8 (9)	5 (16)	1 (3)	2 (6)	
Refused	2 (2)	1 (3)	1 (3)	0 (0)	
Financial well-being or distress, median [IQR] (*n* = 91)^a^	5 [4–6]	5 [4–6]	5 [4–6]	6 [4–6]	
Clinical comorbidity					
Days on hemodialysis, median [IQR]	236 [124–538]	305 [166–520]	198 [87–529]	241 [118–502]	0.5
Comorbidity score, median [IQR]^b^	2 [0–3]	2 [0–4]	1 [0–2]	2 [0–4]	0.04
Comorbidity score tertile					0.03
Lowest, 0 points	30 (33)	8 (26)	11 (37)	11 (35)	
Middle, 1–2 points	32 (35)	9 (29)	15 (50)	8 (26)	
Highest, 3–8 points	28 (30)	13 (42)	3 (10)	12 (39)	
Missing	2 (2)	1 (3)	1 (3)	0 (0)	
Met definition of depression					0.2
No, PHQ-8 score < 10	23 (25)	4 (12.9)	9 (30)	10 (32.26)	
Yes, PHQ-8 score ≥ 10	68 (74)	26 (84)	21 (70)	21 (68)	
Missing	1 (1)	1 (3)	0 (0)	0 (0)	
Depression score, mean (SD)	6(5)	5(3)	6(5)	6(5)	0.9
Dialysis Center Experience					
Time Receiving Care in Dialysis Center					0.9
Less than 3 months	17 (19)	4 (13)	7 (23)	6 (19)	
At least 3 months but less than 1 year	39 (43)	14 (47)	11 (37)	14 (45)	
12 to 18 months	15 (16)	6 (20)	5 (17)	4 (13)	
18 months to 2 years	7 (8)	2 (7)	2 (7)	3 (10)	
Missing	2 (2)	0 (0)	1 (3)	1 (3)	
Dialysis Center Satisfaction Ratings^c^					
Rating of dialysis center staff^c^	9 [8–10]	10 [8–10]	9 [8–10]	8 [7–10]	0.4
Rating of dialysis facility^c^	9 [8–10]	10 [8–10]	9 [8–10]	8 [7–10]	0.3
Advance Preparation for LDKT					
Dialysis initiation					0.9
Planned/dialysis facility	16 (17)	5 (16)	5 (17)	6 (19)	
Planned/hospital	35 (38)	13 (42)	11 (37)	11 (35)	
Urgent/emergency room	41 (46)	13 (42)	14 (47)	14 (45)	
Dialysis initiation with catheter					0.4
No	28 (30)	6 (19)	11 (37)	11 (35)	
Yes	62 (67)	24 (77)	19 (63)	19 (61)	
Don’t know	2 (2.17)	1 (3.23)	0 (0)	1 (3)	
Completed KT evaluation	17 (18)	7 (23)	4 (13)	6 (19)	
Waitlisted for KT					0.6
No	69 (75)	23 (74)	20 (67)	26 (84)	
Yes	16 (17)	5 (16)	7 (23)	4 (13)	
Don’t know	7 (8)	3 (10)	3 (10)	1 (3)	

^a^numbers are n(%) unless otherwise specified; *p* values for comparisons across study groups at baseline ^b^Self-report Charlson comorbidity index weighted for end stage renal disease, scored from 0 to 43 with higher scores indicating greater comorbidity; ^c^Total Score possible 10, with higher scores indicating greater satisfaction

### Participant perceived involvement in kidney treatment decisions at enrollment

Nearly one third of all participants reported they had not talked with any of the doctors, nurses or staff at the hemodialysis facility about kidney transplant. Similarly, nearly one third felt they had not talked to kidney doctors or other treatment staff as much as they wanted about what treatment was right for them over the prior 12 months, and nearly one third reported they were not as involved as they wanted in choosing the treatment for kidney disease that was right for them over the prior 12 months. Perceptions were similar among the three intervention assignment groups.

### Effect of PREPARED interventions on LDKT pursuit, beliefs and concerns

**Fig. 2:**
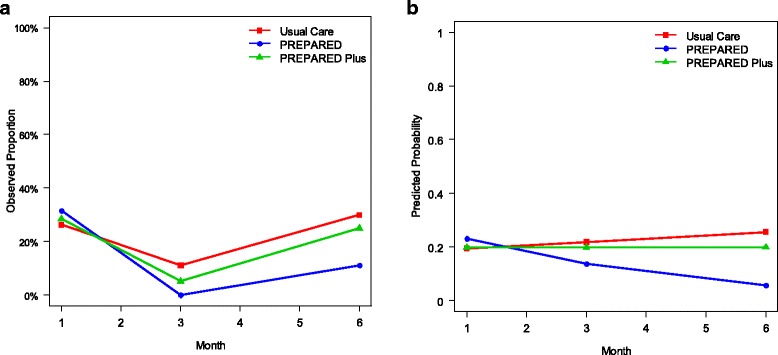
Unadjusted observed proportion (Panel **a**) and adjusted (for participants’ baseline comorbidity scores) predicted probability (Panel **b**) of participants achieving 1 additional live donor kidney transplantation behaviors at 1, 3 and 6 months. In longitudinal GEE analyses, the Odds Ratio (95% CI) for an individual participant achieving at least 1 new behavior over 6 months was 1.53 (0.17, 13.45), 0.15 (0, 5.22), and 1.0 (0.22, 4.68), for participants in the usual care, PREPARED, and PREPARED Plus groups, respectively (*p* = 0.66 in test for global differences across groups in the adjusted analysis)

At enrollment, 27% of all participants reported they had no previous discussions about LDKT with either their doctor or with their family or friends, while 7% had both completed a LDKT evaluation and identified a donor. After six months follow up, 20% of all participants reported they had had no discussions about LDKT with either their doctor or with family or friends, and 16% had completed an LDKT evaluation and identified a donor. The proportion of participants achieving LDKT behaviors was similar across the study groups (Table 2). Within each study group, individual participants experienced no statistically significant change in achieved LDKT behaviors over 6 months, and there was no statistically significant difference in participants’ achievement of LDKT behaviors across study groups (odds ratio [95% confidence interval] for individual achievement of 1 additional LDKT behavior within Usual Care, PREPARED, and PREPARED Plus groups was 1.53 [0.17–13.45], 0.15 [0.0–5.22], and 1.00 [0.22–4.68], respectively, *p* = 0.66 for differences across groups) (Fig. 2). Findings were similar in analyses accounting for potential clustering of participants within their hemodialysis facilities.

**Table 2:** Accomplishment of behaviors reflecting pursuit of LDKT at baseline and 6 months after interventions

	Baseline					6 Months Follow up				
	Overall (*N* = 92)	Usual Care (*N* = 31)	PREPARED (*N* = 30)	PREPARED Plus (*N* = 31)	p*	Overall (*N* = 74)	Usual Care (*N* = 26)	PREPARED (*N* = 22)	PREPARED Plus (*N* = 26)	p*
Behavior Accomplished					0.30					0.9
No behaviors	25 (27)	8 (26)	9 (30)	8 (26)		15 (20)	4 (15)	5 (23)	6 (23)	
Discussed with doctor or family (not both)	20 (22)	4 (13)	5 (17)	11 (36)		17 (23)	6 (23)	5 (23)	6 (23)	
Discussed with doctor and family	21 (23)	10 (33)	8 (27)	3 (10)		16 (22)	5 (19)	6 (27)	5 (19)	
Started evaluation, no donor identified	3 (3)	1 (3)	0 (0)	2 (7)		3 (4)	1 (4)	0 (0)	2 (8)	
Completed evaluation, no donor identified	3 (3)	0 (0)	1 (3)	2 (7)		1 (1)	1 (4)	0 (0)	0 (0)	
Started evaluation, donor identified	14 (14)	6 (19)	4 (13)	4 (13)		10 (14)	5 (19)	2 (9)	3 (12)	
Completed evaluation, donor identified	6 (7)	2 (7)	3 (10)	1 (3)		12 (16)	4 (15)	4 (18)	4 (15)	

*p values for comparisons across study groups at baseline and across study groups at 6 months

**Fig. 3:**
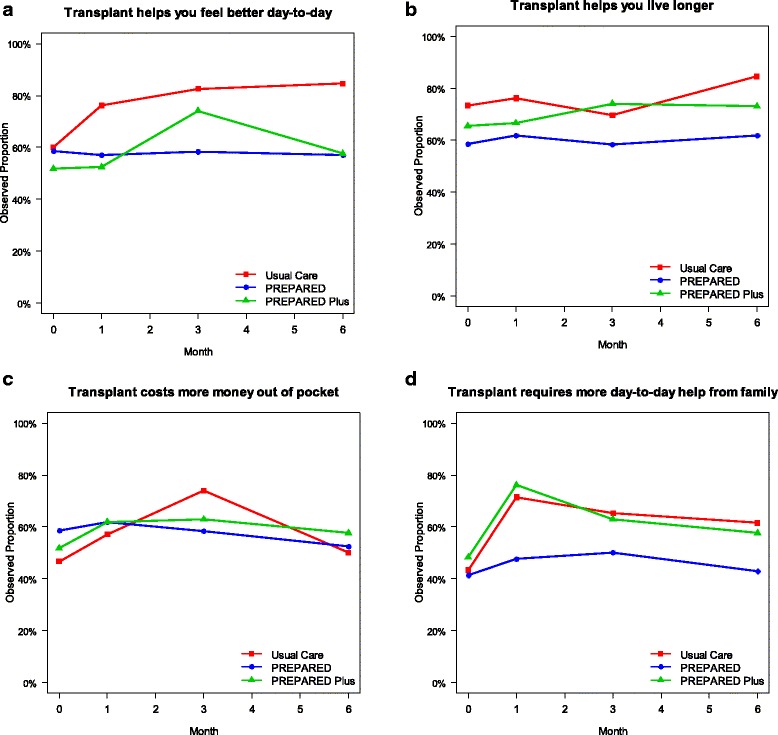
Proportion of participants stating they thought a transplant would help them feel better on a day-to-day basis (panel **a**), help them live longer (panel **b**), cost more money out of pocket than other treatments (panel **c**), or require more day-to-day help from family (panel **d**) at baseline, 1, 3, and 6 months follow up. In generalized estimating equation models, individuals’ beliefs did not statistically significantly change during the study and there were no statistically significant differences between study groups

At enrollment, the majority of all participants reported they felt transplant would help them feel better on a day-to-day basis (*n* = 51, 55%), help them live longer (*n* = 60, 65%), and cost more out of pocket (*n* = 48, 52%) compared to hemodialysis. Fewer than half (*n* = 40, 44%) reported they felt transplant would require more help from their family with taking them to appointments and assisting with daily activities. Participants’ beliefs did not statistically significantly change over 6 months follow up and were not statistically significantly different among study groups in GEE models (Fig. 3). At enrollment, participants overall were most concerned about their family members’ safety after the surgery, their own safety during transplant surgery, feeling guilty or indebted to their family member, and family members’ money matters after LDKT donation. Participants’ beliefs did not statistically significantly change over 6 months follow up and were not statistically significantly different among study groups in GEE models (Table 3).

**Table 3:** Participants’ median [interquartile range] ratings^a^ of potential concerns regarding LDKT at baseline and follow-up

	Baseline					6 Months Follow up				
	Overall (*N* = 92)	Usual Care (*N* = 31)	PREPARED (*N* = 30)	PREPARED Plus (*N* = 31)	p	Overall	Usual Care (*N* = 26)	PREPARED (*N* = 22)	PREPARED Plus (*N* = 26)	p
Need for help after surgery	5 [0–8]	5 [0–8]	4 [0–7]	5 [0–9]	0.54	5 [0–8]	4 [0–8]	5 [0–10]	1 [0–5]	0.15
Future ability to have children	0 [0–8]	0 [0–7]	0 [0–8]	0 [0–6]	0.9	0 [0–4]	0 [0–1]	0 [0–5]	0 [0–0]	0.74
Recipients’ money matters	5 [0–10]	7 [4–10]	5 [0–10]	5 [0–10]	0.2	2 [0–9]	0 [0–7]	5 [0–9]	0 [0–8]	0.51
Family members’ money matters	6 [0–10]	10 [5–10]	5 [0–7]	6 [2–10]	< 0.01	4 [0–10]	5 [0–10]	5 [0–10]	0 [0–7]	0.23
Recipient safety during surgery	8 [5–10]	8 [5–10]	8 [5–10]	8 [5–10]	0.74	6 [0–10]	7 [1–10]	6 [0–9]	6 [0–10]	0.66
Family members’ safety during surgery	10 [6–10]	10 [8–10]	8 [5–10]	10 [6–10]	0.45	8 [5–10]	7 [4–10]	10 [6–10]	8 [3–10]	0.59
Family member feeling pressured or guilty	5 [0–10]	8 [0–10]	5 [0–10]	5 [0–9]	0.57	2 [0–9]	5 [0–9]	4 [0–10]	0 [0–5]	0.41
Recipient feeling guilty or indebted	7 [0–10]	6 [0–10]	6 [1–10]	8 [0–10]	0.85	2 [0–10]	6 [0–10]	5 [0–10]	0 [0–5]	0.08
Concern about relationship with family member donor	3 [0–8]	5 [0–10]	1 [0–6]	3 [0–8]	0.52	0 [0–7]	1 [0–9]	0 [0–5]	0 [0–4]	0.27

^a^Participants rated their concerns from 0 (no concern) to 10 (extremely concerned); In GEE analyses. Individuals’ concerns about LDKT did not statistically significantly change during study follow-up, and there were no statistically significant differences in concerns among study groups

### Intervention fidelity and perceived usefulness

Most participants enrolled in the PREPARED (*n* = 29, 97%) or PREPARED Plus (*n* = 31, 100%) groups met with study staff to receive the interventions. A majority of participants reported the PREPARED video and book were helpful with numerous aspects of informed decision-making (Table 4). Fewer than one third of PREPARED Plus participants reported sharing the financial intervention with a potential donor at 1 (*n* = 6, 27%), 3 (*n* = 8, 30%), or 6 (*n* = 6, 24%) months after enrollment. None of the participants enrolled in PREPARED Plus arm utilized the living donor financial assistance program.

**Table 4:** Participants’ views on helpfulness of informational materials regarding treatment decision-making 1 month, 3 months, and 6 months after enrollment

	1 month		3 months		6 months	
	PREPARED *N* = 22 n(%)	PREPARED Plus *N* = 21 n(%)	PREPARED *N* = 24 n(%)	PREPARED PLUS *N* = 27 n(%)	PREPARED *N* = 22 n(%)	PREPARED Plus *N* = 26 n(%)
PREPARED video and book helped^a^ participants to:						
Think about treatment choices	16 (73)	11 (52)	16 (67)	21 (78)	16 (73)	13 (52)
Prepare to make a better decision about future kidney disease treatments	16 (73)	13 (62)	12 (50)	19 (70)	14 (64)	14 (56)
Think about the pros and cons of different treatment options in the future	16 (73)	13 (62)	17 (71)	18 (67)	15 (68)	14 (56)
Think about which pros and cons are most important	15 (68)	14 (67)	15 (63)	18 (67)	15 (68)	14 (61)
Know that the choice depends on what matters most to them	17 (77)	13 (62)	15 (63)	19 (70)	16 (73)	17 (68)
Organize their thoughts about the choice	14 (64)	14 (67)	13 (54)	19 (70)	14 (64)	14 (56)
Think about how involved they want to be in the decision	16 (73)	14 (67)	16 (67)	20 (74)	17 (77)	17 (68)
Identify questions to ask their doctor	13 (59)	13 (62)	15 (63)	18 (67)	16 (73)	15 (60)
Prepare to talk to their doctor about what matters most to them	13 (59)	14 (67)	16 (67)	18 (67)	16 (73)	15 (60)

^a^Proportion of participants saying the PREPARED video and book helped them “quite a bit” or “a great deal” with decision making aspects listed (versus “somewhat,” “a little,” or “not at all”

## Discussion

In this small clinical trial, we found that providing informational decision aids and offering donor financial assistance to African Americans receiving in-center hemodialysis did not appear to increase their pursuit of LDKT over 6 months, despite participants’ desires to be more involved in their LDKT decisions and their positive beliefs about LDKT. Interventions also had no effect on participants’ views of the potential benefits of LDKT or their concerns about LDKT. Participants found informational decision aids helpful in thinking about and making decisions about their treatment choices, but they were no more likely to initiate LDKT discussions with their families or doctors or to complete other key LDKT behaviors (e.g., transplant evaluation). Further, participants who were offered the donor financial assistance program did not utilize it. These findings provide important preliminary evidence regarding the deployment of these interventions in clinical settings as well as considerations for their study in future large scale studies.

To our knowledge, this is the first rigorously conducted trial to study the effect of providing informational decision aids or offering donor financial assistance on potential African American recipients’ pursuit of LDKT. A previous observational study suggested education could increase patients’ readiness to pursue LDKT, especially among women and other subgroups, but this study did not employ a comparison group or explore effects of education longitudinally [30]. Another observational study demonstrated that African Americans presenting to a transplant center with higher levels of transplant knowledge were more likely to receive LDKT within 1 year [31]. However this study also did not randomly assign educational interventions and could have been subject to residual confounding, particularly if those with greater transplant knowledge had other types of resources available to them that were not accounted for in the study design. We are aware of no prior study of the effectiveness of a living donor financial support intervention to improve pursuit of LDKT among African American hemodialysis patients.

Consistent with other studies, we found that many African American patients reported they started hemodialysis urgently or emergently [32–35], had not completed a transplant evaluation within 1–2 years of initiating in-center hemodialysis, and nearly one third could not recall speaking to medical staff about the kidney transplant process [36, 37]. The practice of providing educational materials to patients about kidney transplant is widespread and advocated as a key mechanism to guide patients toward transplant [18]. However, it is unclear when patients most commonly receive education in relation to dialysis initiation. Although dialysis facilities are required to discuss transplantation as a treatment option, the extent to which patients initiating hemodialysis actually receive or appreciate these discussions has been poorly studied. It is also unclear whether patients who have established long term relationships prior to dialysis are more likely to receive information from nephrologists and to be motivated to seek transplant. Since many study participants reported they initiated dialysis urgently, it is possible they had limited opportunities to discuss LDKT. Our study did not explore whether circumstances surrounding patients’ dialysis initiation or patients’ relationships with their nephrologists could influence their willingness to seek LDKT. We also did not explore reasons why patients had not discussed LDKT with their physicians. Studies exploring these potential influences on patients’ willingness to seek transplant are warranted.

Our findings ran contrary to our a priori hypotheses. Although a majority of participants reported they desired more engagement in treatment decisions and found PREPARED informational materials helpful, it is possible potential LDKT recipients may require different or adjunctive support beyond self-directed education to act on LDKT decisions. Other studies suggest that the effect of decision aids on changing patients’ actual health behaviors may be weak, particularly if health care providers do not assist with decision making or patient activation [38, 39]. Previous trial findings suggest interventions in which transplant teams or lay people directly engage potential transplant recipients to help them consider LDKT, reach out to potential donors [40–42], and navigate patients [43] to complete transplant evaluations are effective in helping patients pursue and obtain LDKT. Further, PREPARED interventions did not provide support for certain LDKT behaviors we measured, including support for talking to their family members or transplant professionals about LDKT. A prior trial demonstrated that interventions (including educational materials and social worker support) providing patients support to talk about LDKT with family members and health care professionals improved these behaviors [42]. Many of our participants were unemployed or disabled, and they may have benefitted from assistance to complete complex processes related to LDKT. Potential LDKT recipients may also have needed more direct assistance to take advantage of the LDKT financial assistance program (e.g., via more intensive financial counseling). In our own prior qualitative studies, potential LDKT recipients reported feeling awkward discussing the potential financial risks of LDKT with potential donors and they cited the value of professional assistance from others (e.g., social workers) to aid with this aspect of the LDKT process [15, 44].

It is worth noting that PREPARED informational decision aids were intended to promote patients’ informed decisions to choose renal replacement therapies that are aligned with their personal values, a goal that is defined as essential to patient centered care [45, 46]. Thus PREPARED materials did not explicitly encourage LDKT as a preferred treatment choice, but instead presented the benefits and risks LDKT in the context of the benefits and risks of other potential treatment options (including dialysis and conservative management) [22]. It is possible that study participants not seeking LDKT in our study made values-based decisions to remain on in-center hemodialysis. Prior studies have shown patients newly initiating in-center hemodialysis often undergo significant physical and psychological adjustments that could interfere with their capacity to make major behavioral changes [47]. It is also important to note that PREPARED did not focus specifically on potential LDKT donors’ outcomes or safety of the LDKT procedure. Since many participants expressed concerns about the impact of LDKT on safety and on donors, information may not have addressed key concerns which could hinder participants’ willingness to pursue LDKT. Notably, a majority of study participants had adequate (i.e., >9th grade) health literacy, suggesting that health literacy was not a barrier to participants’ interpretation of informational materials.

Limitations of our study include its conduct among a small number of African Americans in a single geographic region. Since we did not meet our planned recruitment goal, it is possible our study was underpowered to detect smaller effects of our interventions on pursuit of LDKT. A small proportion of our participants had already completed LDKT evaluations or identified a donor at baseline, which may have further limited our capacity to detect improvements in LDKT pursuit among the study population. Further, only a small subset of participants who were interested in LDKT at baseline would have been eligible to pursue the financial assistance intervention, limiting our capacity to assess this intervention. We only followed participants for 6 months. It is possible patients require substantial time to accomplish LDKT behaviors, particularly when have recently initiated in-center hemodialysis, and are likely to experience substantial physical and psychological adjustment to their treatment [48–51]. For patients who are just initiating dialysis and may have limited exposure to transplantation, longer follow up (e.g., up to 10 years) may be needed to assess the long term impact of PREPARED interventions. We also did not measure changes in general transplant-decision-making or transplant knowledge as primary outcomes, which could also be important indicators of willingness to seek LDKT. Importantly, we also did not collect information on the education provided to participants in the course of their usual care. While all three intervention groups would have received this information, it is possible that PREPARED information was not viewed as complimentary to usual education or that usual education varied across dialysis centers. Behaviors toward LDKT have not been validated as a strong predictor of receiving LDKT. Measures to validate behaviors toward LDKT should be developed and could be deployed in future studies. Finally, it is possible participants who received the intervention could have shared the video and the book with others in the dialysis facility, contaminating our findings. However, we advised participants not to share the interventions with others and participants in the usual care group reported they had not seen the video or the book. Finally, although we believe patients received standard of care, it is possible participants’ behaviors were influenced by practice patterns (e.g., transplant referrals processes) within their dialysis facilities. Despite these limitations, this study is among the first to quantify the impact of broadly supported interventions to improve LDKT and provides important insight to guide future interventions. Larger studies with longer observation periods may be needed to more accurately assess the value of these interventions to address LDKT disparities.

## Conclusions

In summary, informational decision aids and living donor financial support interventions were viewed favorably by African American potential LDKT recipients but did not improve their pursuit of LDKT in a small preliminary trial. Larger studies designed with longer follow up and deploying these interventions in ways that might better facilitate their uptake to improve African Americans’ receipt of LDKT are urgently needed.

## Abbreviations

CAHPS®: Consumer Assessment of Healthcare Providers and Systems
GEEs: Generalized Estimating Equations
LDKT: Living Donor Kidney Transplants
PREPARED: Providing Resources to Enhance African American Patients’ Readiness to Make Decisions about Kidney Disease
